# Nitric Oxide Bioavailability in Obstructive Sleep Apnea: Interplay of Asymmetric Dimethylarginine and Free Radicals

**DOI:** 10.1155/2015/387801

**Published:** 2015-05-06

**Authors:** Mohammad Badran, Saeid Golbidi, Najib Ayas, Ismail Laher

**Affiliations:** ^1^Department of Pharmacology and Therapeutics, Faculty of Medicine, University of British Columbia, Vancouver, BC, Canada V6T 1Z3; ^2^Divisions of Critical Care and Respiratory Medicine, Department of Medicine, 2775 Laurel Street, 10th Floor, University of British Columbia, Vancouver, BC, Canada V5Z 1M9; ^3^Sleep Disorders Program, UBC Hospital, Room G-285, 2111 Wesbrook Mall, Vancouver, BC, Canada V6T 2B5; ^4^Division of Critical Care Medicine, Providence Health Care, St. Paul's Hospital, Vancouver, BC, Canada V6Z 1Y6

## Abstract

Obstructive sleep apnea (OSA) occurs in 2% of middle-aged women and 4% of middle-aged men and is considered an independent risk factor for cerebrovascular and cardiovascular diseases. Nitric oxide (NO) is an important endothelium derived vasodilating substance that plays a critical role in maintaining vascular homeostasis. Low levels of NO are associated with impaired endothelial function. Asymmetric dimethylarginine (ADMA), an analogue of L-arginine, is a naturally occurring product of metabolism found in the human circulation. Elevated levels of ADMA inhibit NO synthesis while oxidative stress decreases its bioavailability, so impairing endothelial function and promoting atherosclerosis. Several clinical trials report increased oxidative stress and ADMA levels in patients with OSA. This review discusses the role of oxidative stress and increased ADMA levels in cardiovascular disease resulting from OSA.

## 1. Obstructive Sleep Apnea

**Figure 1:**
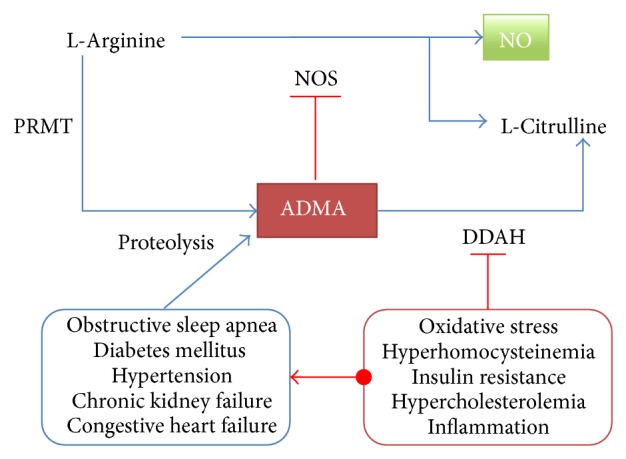
Pathology of ADMA. ADMA: asymmetric dimethylarginine; DDAH: dimethylarginine dimethylaminohydrolase; NO: nitric oxide; NOS: nitric oxide synthase; PRMT: protein arginine methyltransferase.

Obstructive sleep apnea (OSA) is a sleep-breathing disorder characterized by momentary episodes of either 80–100% reductions in airflow for periods of ten seconds or more (apnea) or 50–80% reductions (hypopnea) caused by a collapsed or obstructed upper airway; these two conditions can lead to hypoxemia (low levels of oxygen in blood) and hypercapnia (high levels of carbon dioxide in blood). Patients are categorized as having mild, moderate, or severe OSA depending on the apnea/hypopnea index (AHI), which is defined as the total numbers of obstructive apnea/hypopnea episodes per hour of sleep. In normal individuals the index is usually 5 or lower, while it is 5–15 in mild, 15–30 in moderate, and 30 or more in severe OSA patients [1, 2]. In patients with mild OSA the oxyhemoglobin saturation drops to 95% and can drop below 80% in severe cases. Obstruction of the airways results in greater breathing effort and a more negative intrathoracic pressure, resulting in arousal, sleep interruption, and reopening of the airways [3] as summarized in Figure 1.

Risk factors for sleep apnea include obesity, craniofacial abnormalities, smoking, male gender, short neck, and menopause in women. Obesity is one of the main risk factors of sleep apnea since 60% to 90% of OSA patients are obese and there is a positive correlation between body mass index (BMI) and OSA [4, 5]. The overlap of obesity and OSA poses a challenge to ascribing the relative contributions of these comorbidities to cardiovascular complications. Greater adiposity and a shorter neck add weight to the soft tissue within the upper airway and the neck, so further increasing airway collapsibility [6]. The economic burden created by OSA is considerable; for example, OSA-related automobile collisions in the year 2000 alone are attributed to 1400 fatalities at a total cost of 15.9 billion dollars in the USA. It is estimated that treatment with continuous positive airway pressure (CPAP) produced a saving of 7.9 billion dollars and 1000 lives [7].

Between 3.7% and 26% of the population has an AHI above 5. The prevalence of OSA, defined mainly by AHI frequency and the presence of hypersomnolence, is estimated to range from 1.2% to 7.5%. Due to the lack of homogeneity in these epidemiologic studies, estimates show wide variations. For instance, some studies were performed in preselected population groups while others included a high number of subjects who were suspected of having OSAS because of their snoring frequency. Moreover, some earlier studies did not include subjects over 60 years of age [8]. Unfortunately, most of those who are affected by OSA remain undiagnosed despite medical advances [9].

The results of several clinical studies strongly suggest that OSA is an independent risk factor for cardiovascular diseases such as hypertension, coronary artery disease, stroke, and heart failure [10–17]. Several mechanisms are thought to link OSA and vascular diseases, including increases in sympathetic activation, oxidative stress, inflammation, endothelial dysfunction, coagulation, and metabolic dysregulation [18]. We review the bioavailability of nitric oxide (NO) metabolism in OSA and the role of asymmetric dimethylarginine (ADMA) as a risk factor for endothelial dysfunction.

## 2. ADMA and Nitric Oxide Metabolism

The 1998 Nobel Prize in Medicine and Physiology was awarded for the discovery of NO as a signaling molecule in the cardiovascular system [19]. This gaseous vasodilator has a half-life of 2–30 sec [20] and is synthesized from the amino acid L-arginine in endothelial cells by the calcium-calmodulin dependent enzyme nitric oxide synthase (NOS) [21, 22]. It was later found that NO relaxes smooth muscles despite the high levels of calcium and activated myosin, most likely via NO-mediated heat shock protein 20 (HSP20) phosphorylation [22, 23]. NO diffuses in blood where it binds to hemoglobin and is then excreted in urine as nitrate after being oxidized. Vasodilation is produced when NO stimulates soluble guanylate cyclase, leading to increased production of cyclic guanosine monophosphate (GMP) that activates GMP-dependent kinases to decrease intracellular calcium concentrations [24]. NO is also antithrombotic, antiproliferative, and anti-inflammatory [25–33].

ADMA is a naturally occurring L-arginine analog derived from the proteolysis of methylated proteins [34]. The terminal guanidine group of arginine is demethylated by two classes of protein arginine methyltransferases (PRMTs). Type 1 PRMTs catalyze the formation of ADMA, whereas type 2 PRMTs lead to the formation of symmetric dimethylarginine (SDMA) [35]. ADMA inhibits all three isoforms of nitric oxide synthase (NOS) producing NO (neuronal NOS “nNOS,” inducible NOS “iNOS,” and endothelial NOS “eNOS”) but with different affinities; for example, ADMA has an IC_50_ value of 10 *μ*mol/L in cultured endothelial cells [36] but low affinities for nNOS and iNOS (*K*_*i*_ > 300 *μ*mol/L) [37]. ADMA is both exported from its site of origin (such as liver, kidney, or lung) and imported from the plasma at distant sites via cationic amino acid transporters (CATs) in exchange for arginine and other cationic amino acids (CAAs) [38]. While ADMA is present in the liver and kidneys, the lungs produce 4 times more [39, 40]. ADMA competes with L-arginine for the binding site in the active center of NOS enzymes [41]. Furthermore, ADMA can “uncouple” NOS by shifting the balance from NO generation to superoxide production such as formation of reactive oxygen species (ROS) [42]. The role of SDMA in the endothelial NO pathway remains unclear. SDMA and ADMA are able to interfere with the substrate availability of NOS by inhibiting the transmembrane cationic amino acid transport (CAT) system of L-arginine, but the IC_50_ values (ADMA and SDMA inhibited CAT1-mediated uptake of L-arginine with IC_50_ values of 758 (460–1251) *μ*mol/L and 789 (481–1295) *μ*mol/L, resp.) are above the estimated endogenous ADMA and SDMA concentrations (13.5 ± 0.13 *μ*mol/L) [43]. Almost 80% of ADMA is enzymatically hydrolyzed by dimethylarginine dimethylaminohydrolase (DDAH), which is expressed in two isoforms: DDAH-1 and DDAH-2, which have distinct tissue distribution and are encoded by different genes and, possibly, exhibit distinct functional roles [44, 45]. The *K*_*m*_ of DDAH is approximately 180 *μ*mol/L and is much higher than normal intracellular concentrations, which enables the enzyme to properly accomplish this task. This ensures that under normal conditions the enzyme performs in the linear part of the substrate-velocity curve, meaning that the rate of ADMA degradation is approximately proportional to its concentration. The activity of DDAH increases with higher ADMA levels, thus preventing accumulation of ADMA. The high *K*_*m*_ of DDAH prevents accumulation of high ADMA levels and ensures sufficient amounts of ADMA are available to function as a regulator of NOS activity [46].

Elevated ADMA levels occur in patients with chronic kidney failure and were proposed to act as possible endogenous inhibitors of NO synthesis by Vallance et al. [47]. Plasma levels of ADMA predicted all causes of mortality, but not cardiovascular disease incidence, in the Framingham Offspring Study of 3320 normal subjects followed up for 11 years [48]. The many studies on ADMA have led many to propose that it alters NO biosynthesis and mediates several cardiovascular complications as summarized in Table 1 [49]. ADMA levels are elevated in patients with hypertension [50], diabetes mellitus [51], coronary artery disease [21], arrhythmias [52], heart failure [53], and cerebrovascular disease [54].

Bae et al. measured plasma ADMA levels in 48 patients newly diagnosed with acute coronary syndrome (ACS) and followed changes in ADMA concentrations during their medical therapy which included a combination of drugs with or without percutaneous coronary interventions. Concentrations of plasma ADMA in ACS patients were double that in age-matched control patients, but levels decreased significantly after 2 weeks of treatment [55]. Levels of ADMA were also significantly elevated in 198 hemodialysis patients with left ventricular hypertrophy (LVH) (3.00 *μ*mol/L with LVH and 1.88 *μ*mol/L without LVH) and were correlated with left ventricular mass (*r* = 0.26, *P* < 0.001) [56]. Levels of ADMA are also increased after cardioembolic infarction and transient ischemic attack (TIA) in 363 CVD patients when compared to 48 control patients [57]. Results of other studies on ADMA in CVD are discussed in Table 1.

## 3. ADMA in OSA

It is now well established that OSA is a cardiovascular risk factor and that ADMA has the potential to exacerbate cardiovascular disease. But does OSA influence ADMA levels? Many studies show increases in ADMA levels in OSA patients; Barceló et al. measured plasma ADMA in 23 OSA patients and found it to be significantly higher (1.17 *μ*mol/L) compared to control (0.87 *μ*mol/L, *P* < 0.01) [61]. Ozkan et al. also reported nonsignificant increases in ADMA levels in OSA patients [62]. Treatment with CPAP for 4 weeks lowered ADMA levels in patients with OSA while also improving forearm mediated dilation (FMD) (0.22 ± 0.27 mol/L before CPAP, 0.21 ± 0.44 mol/L at 1 week, and 0.16 ± 0.27 mol/L at 4 weeks after CPAP, *P* = 0.054) [63]. It is unclear how OSA increases ADMA levels but it is important to note that CPAP treatment can decrease its levels.

## 4. Oxidative Stress in OSA

Reactive oxygen and nitrogen species (ROS, RNS) are normal products of cellular metabolism labeled as molecules or molecular fragments. They contain one or more unpaired electrons in their molecular or atomic orbitals. At low concentrations, these species play an important role in cellular signaling, specifically in defense against infectious agents and induction of mitogenic response [64]. Overproduction of ROS/RNS or deficiency of enzymatic and nonenzymatic antioxidants can lead to oxidative and nitrosative stress. Consequently, oxidative stress leads to an imbalance between prooxidant/antioxidant reactions related to oxygen metabolism. ROS can oxidize lipids, protein, or DNA, so inhibiting their function and disturbing many cellular processes [64]. As a result, oxidative stress has been implicated in many human diseases (e.g., cardiovascular diseases, cognitive impairment, and diabetes) [65–67] as well as in the aging process [68].

Intermittent hypoxia (IH), a hallmark of OSA, is characterized by repeated episodes of hypoxia interspersed with episodes of normoxia, potentially similar to the ischemia/reperfusion (I/R) that results in injury due to the burst of ROS production during the reperfusion period [69]. It is well established that oxidative stress is a major consequence of I/R injuries [70]. Similarly, cycles of IH in OSA patients promote ROS generation and oxidative stress through comparable pathways and various sources, as described in the next section.

## 5. Sources of ROS/RNS in OSA

During aerobic respiration, approximately 3–5% of the oxygen consumed by the mitochondria is converted to superoxide anion, a major form of ROS. Due to hypoxia, there is an increase in ROS production as a result of excessive mitochondrial reduction [64, 71, 72]. Numerous clinical and animal studies demonstrate that IH is correlated with mitochondrial dysfunction, leading to oxidative stress [73–76]. Another important source of ROS in OSA is NADPH oxidase, primarily expressed in leukocytes and activated during inflammatory processes such as infections, where it produces superoxide anions to destroy pathogens. There is increased production of ROS in stimulated neutrophils and monocytes from OSA patients as shown by Schulz et al. [77]. Other studies, however, reported high ROS production even in nonstimulated neutrophils and monocytes [78, 79]. Recent studies by Loffredo et al. assessed FMD in children with OSA and found that it is lower when compared to control and it was inversely correlated with serum soluble NOX2-derived peptide (sNOX2-dp). After adenotonsillectomy, however, FMD was significantly increased and sNOX2-dp was significantly decreased. These data suggest that NOX2-derived oxidative stress is associated with arterial dysfunction in children with OSA [80]. In addition, animal studies show that NADPH oxidase is activated in many tissues such as the brain and carotid body in response to IH [81, 82]. Other sources of ROS that have been extensively studied in IH and OSA are xanthine oxidase [83, 84] and uncoupled eNOS [85].

## 6. Markers of Oxidative Stress in OSA

Throughout the last decade, there has been accumulation of much evidence that linked OSA and IH to oxidative stress. Oxidative stress markers such as lipid peroxidation, protein carbonylation, and DNA oxidation are prominent in OSA patients and animals subjected to IH. Patients with severe OSA have higher urinary 8-isoprostane levels, which then decreased after 6 months of CPAP therapy [86]. In a different study, TBARS levels were significantly higher in severe OSA patients after overnight fasting when compared to control [87]. In contrast, Svatikova et al. measured TBARS, oxidized LDL, and isoprostanes in healthy OSA patients with no comorbidities and found no association between severity of OSA and oxidative stress markers [88]. They suggested that, in the absence of significant comorbidities, sleep apnoea does not, in and of itself, initiate the generation of oxidative stress or lipid peroxidation. However, it is possible that, in the setting of comorbidities such as hypertension, vascular disease, and the metabolic syndrome, the oxidative consequences of sleep apnoea may become apparent. In another study, Vatansever et al. measured protein carbonyl and adiponectin levels in patients with mild, moderate, and severe OSA. They found that adiponectin levels were significantly decreased while protein carbonyl levels were significantly elevated in moderate to severe OSA patients but not in mild OSA ones when compared to control [89]. Yamauchi et al. measured another marker of oxidative related DNA damage, 8-OHdG, in OSA patients. They compared 8-OHdG levels between patients with nonsevere OSA (AHI < 30) and patients with severe OSA (AHI > 30) and found that it was higher in severe OSA patients. They also found that 8-OHdG is significantly correlated with AHI, oxygen desaturation index (ODI), and duration of oxygen saturation <90%. However, after adjusting for confounding factors, only ODI was significantly correlated to ODI [90].

## 7. Endothelial Dysfunction in OSA

Endothelial dysfunction manifests as an imbalance between vasodilating and vasoconstricting substances produced by or acting on the endothelium. It is clinically relevant in OSA even in subjects with no history of vascular disease or comorbidities [91]. Decreased endothelium-dependent vasodilation has been reported in several studies using FMD. For example, Kato et al. reported that OSA patients had blunted response to acetylcholine while responses to sodium nitroprusside or verapamil were unaltered [92]. Other studies analyzed nitrite/nitrate levels as a measure of circulating NO levels and reported reductions in OSA subjects [91, 93]. However, CPAP treatment of OSA patients restores circulating NO levels and the levels of its substrate L-arginine [94] as well as FMD [95].

Suggested mechanisms for endothelial dysfunction include (1) interaction between NO and superoxide anion leading to increased formation of a highly unstable RNS peroxynitrite, (2) decreased expression and/or uncoupling of endothelial nitric oxide synthase (eNOS), and (3) increased levels of endogenous eNOS inhibitors such as ADMA [1]. The large volume of distribution and short half-life of peroxynitrite could account for similar levels of nitrotyrosine in OSA and healthy subjects [96, 97]. Microcirculatory endothelial cells (EC) from OSA patients have increased formation of peroxynitrite [98]. In cultured endothelial cells from OSA patients, Jelic and le Jemtel reported a significant decrease in both total and phosphorylated eNOS levels, which were restored by CPAP treatment [99]. Moreover, Tanaka et al. suggested that eNOS activation is regulated by redox status and that increased oxidative stress reduced eNOS activity by suppressing its phosphorylation [100]. Nonetheless, the expression and activity of eNOS have been reported to be upregulated, [101, 102] downregulated, [103, 104], or unchanged [105] in various experimental models of hypoxia and repetitive hypoxia/reoxygenation. Interestingly, Kaczmarek et al. showed that cultured endothelial cells originating from distinct vascular beds in OSA patients and mice responded differently to IH stress in terms of eNOS expression [106]. Another cause of endothelial impairment is the eNOS uncoupling. For production of NO to occur, five cofactor groups (FAD, FMN, heme, Ca^2+^-calmodulin, and BH_4_) are needed to incorporate oxygen in L-arginine. A lack of any of these cofactors leads to production of superoxide anion instead of NO and adds insult to injury [18]. For instance, increased ROS production, especially superoxide anion during hypoxia, can lead to the oxidation of BH_4_ to BH_2_ rendering eNOS in an uncoupled state as shown by Antoniades et al. [107]. They also reported that increased levels of arginase II degrade L-arginine, leading to further eNOS uncoupling. A recent study by Varadharaj et al. reported that eNOS dysfunction in OSA patients was reversible with BH_4_ treatment [108].

## 8. ADMA in OSA

**Figure 2:**
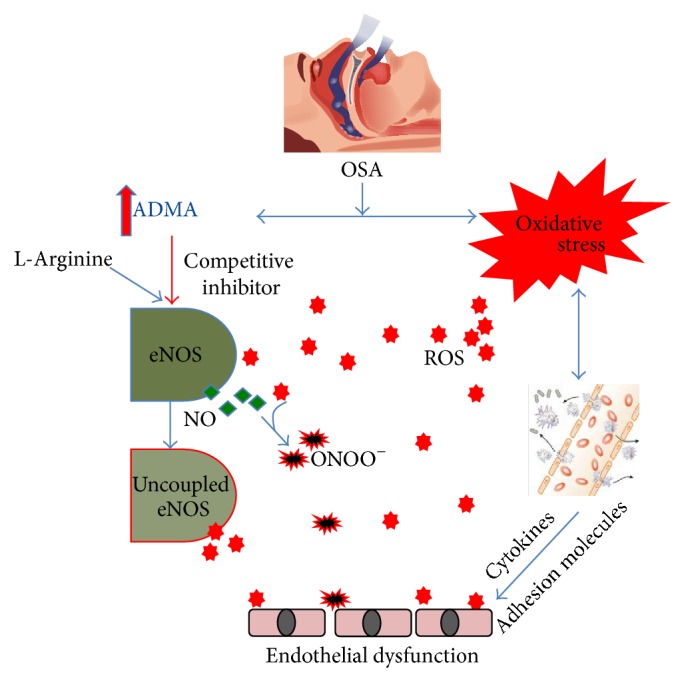
Oxidative stress and high ADMA levels can cause endothelial dysfunction in OSA. ADMA: asymmetric dimethylarginine; eNOS: endothelial nitric oxide synthase; NO: nitric oxide; ONOO^−^: peroxynitrite; OSA: obstructive sleep apnea; ROS: reactive oxygen species.

A recent meta-analysis of 22 studies with a total of 6168 patients reported a correlation between ADMA levels and carotid intima-media thickness (pooled correlation coefficient of 0.29; *P* < 0.001) [109]. This relation was stronger in patients with chronic kidney disease (CKD) than in subjects with normal kidney function. Patients with essential hypertension have impaired FMD and increased serum ADMA levels when compared to controls (0.59 ± 0.14 *μ*mol/L versus 0.40 ± 0.09 *μ*mol/L, *P* < 0.0001); these measures independently accounted for 33.9% of the interindividual variability in peak FMD [110]. In another cross-sectional study of 121 nondiabetic patients with proteinuria, ADMA levels were independently related to FMD (>2.76 *μ*mol/L; *r* ^2^ = 0.27, *P* = 0.0002) and were correlated with both proteinuria (>2.5 g/d; *r* ^2^ = 0.40, *P* < 0.001) and the presence or absence of secondary amyloidosis (*r* ^2^ = 0.42, *P* = 0.0003) [111]. The Cardiovascular Risk in Young Finns Study, a follow-up study of cardiovascular risk from childhood to adulthood, used ultrasound to measure FMD both in 2001 and 2007 in 1808 healthy subjects aged 24–39 years at baseline. Using a multivariable model adjusted with brachial diameter and conventional cardiovascular risk factors, the study reported that baseline ADMA levels were inversely associated with FMD measured six years later (*β* ± SE: −1.89 ± 0.69%, *P* = 0.006). This data suggests that plasma ADMA can predict endothelial function in subjects with no atherosclerotic disease, suggesting that ADMA may be a useful biomarker of endothelial dysfunction and atherosclerosis progression [112], as illustrated in Figure 2. OSA and COPD are among the most common pulmonary diseases, such that many patients have both disorders; this “overlap syndrome” causes more severe nocturnal hypoxemia than either disease alone. ADMA levels were measured in these patients before and after CPAP and there was no significant difference (0.58 ± 0.10 versus 0.61 ± 0.12, *P* = 0.32) despite the reduction in serum levels of other inflammatory factors such as CRP (0.83 ± 0.95 versus 0.53 ± 0.56, *P* = 0.02) [76, 113]. Barcelo et al. investigated the influence of OSA on the diurnal variations in some markers of endothelial dysfunction such as ADMA and soluble CD40 ligand and found that ADMA levels were significantly related to arousal index (*P* = 0.046). They also suggested that ADMA levels might be dependant either on obesity index or metabolic dysfunction rather than on OSA alone [114]. In nonobese children with OSA, Gozal et al. reported no differences in ADMA levels compared to matched controls [0.79 ± 0.20 *μ*mol/L in OSA children (*n* = 46) and 0.87 ± 0.19 *μ*mol/L in controls (*n* = 22;* p* = NS)]. Soluble CD40 ligand levels were higher in OSA compared with controls (15,128 ± 597 pg/mL versus 5729 ± 653 pg/mL, *P* < 0.00001) and were reduced after treatment (adenotonsillectomy) (9866 ± 702 pg/mL, *P* < 0.0002) [115].

## 9. ADMA as a Treatment Target

Treatment with CPAP decreases ADMA levels and improves endothelial function. Another option would be supplementation with L-arginine, since it can displace ADMA from its receptor and also improve NO bioavailability despite the fact that its plasma concentration is 25 times in excess of it's *K*_*m*_ for eNOS [36, 116]. Unfortunately, L-arginine is ineffective after long-term use and, even worse, is correlated with mortality when given to post-MI patients [117, 118]. Other drugs used to lower ADMA levels include statins, angiotensin converting enzyme inhibitors (ACEI), *β*-blockers, aspirin, and antioxidants such as vitamins E and C. These have largely been used for short periods in small groups of patients [119]. Large-scale clinical trials are needed to evaluate the usefulness of these drugs in OSA patients. The therapeutic regulation of ADMA via DDAH is another possible mechanism to increase NO bioavailability [120, 121]. The clinical relevance of regulating the endothelial ADMA/NO pathway in patients with OSA and oxidative stress induced by ADMA may be a useful strategy pending the availability of selective DDAH regulators.

## 10. Conclusion

There is extensive support for the role of ADMA as a regulator of NO production by the vascular endothelium. Data from cell culture and animal experiments and cross-sectional studies in humans suggest an association between elevated ADMA concentrations and cardiovascular diseases. Elevation of ADMA levels, possibly in combination with increases in superoxide levels in patients with OSA, reduces the bioavailability of NO and leads to endothelial dysfunction to promote atherosclerosis and cardiovascular disease. Prospective clinical studies suggest that ADMA may be a potential diagnostic tool for cardiovascular risk assessment, but the use of ADMA in currently applied risk scores remains untested.

To date, pharmacological strategies targeting ADMA in cardiovascular risk reduction have been disappointing, as most lipid lowering and blood pressure-lowering drugs did not change ADMA concentrations significantly. Currently, only treatment with CPAP has been shown to normalize ADMA levels in OSA patients. Specific therapeutic interventions of DDAH/ADMA metabolism are needed to perform randomized controlled trials to assess the clinical benefits of modulating ADMA levels in OSA patients.

## Figures and Tables

**Table 1:** ADMA as a cardiovascular risk marker in some cardiovascular clinical studies.

CVD	AMDA levels (*μ*mol/L)	Result	Reference
Stable CAD	>0.62 (highest quartile)	3.9-fold increased risk of acute coronary disease	[58]
MI	1.45 (median range of the highest tertile)	ADMA predicts mortality after MI	[59]
CHF	>2.5 (highest tertile)	High ADMA levels are associated with cardiac decompensation and major adverse cardiovascular events	[60]
AF	>0.63 (cut-off value)	High ADMA levels predict AF recurrence after catheter ablation	[52]
Ischemic stroke	>1.43 (above 90th percentile)	High ADMA levels are associated with increased risk of stroke in old patients and in patients with hyperhomocysteinemia	[54]

ADMA: asymmetric dimethylarginine; AF: atrial fibrillation; CAD: coronary artery disease; CHF: congestive heart failure; MI: myocardial infarction.
